# Attitudinal professionalism under administrative embedding: evidence from social workers in urban China

**DOI:** 10.3389/fpsyg.2025.1624360

**Published:** 2025-12-10

**Authors:** Ziyu Liu, Hung Wong

**Affiliations:** 1 Department of Sociology, Jimei University, Xiamen, China; 2 School of Social Work, Chinese University of Hong Kong, Hong Kong, Hong Kong SAR, China

**Keywords:** attitudinal professionalization, organization embedding, value-discretion divergence, Chinese social workers, professional socialization

## Abstract

**Introduction:**

Attitudinal professionalism—comprising individual values, role orientations, and perceived discretion—is central to understanding social workers' professional behavior. In China, social work operates within an administratively embedded service regime shaped by government purchase-of-service contracts, raising questions about how organizational embedding and socialization processes influence professionals' value orientations and perceived autonomy. This study examines the multidimensional structure of attitudinal professionalism among urban social workers and the factors associated with its variation.

**Methods:**

An online survey was administered to 667 frontline social workers in Guangzhou, Shenzhen, and Shanghai. Five attitudinal dimensions were measured: professional organization reference, public service, self-regulation, sense of calling, and perceived autonomy. Multiple regression analyses were conducted to estimate associations with professional socialization indicators (education, certification, tenure, gender) and organizational embedding, operationalized as organizational dependence on government purchase-of-service contracts.

**Results:**

Respondents demonstrated strong commitments to public service and calling but lower levels of perceived autonomy, indicating a value-discretion divergence consistent with managerial and contractual service logics. Organizational independence—lower reliance on government contracts—was positively associated with value-oriented dimensions. In contrast, higher educational attainment and formal certification were negatively associated with certain professionalism dimensions, suggesting exam-centric training pathways and uneven practice-integrated socialization. Overall, occupational value orientations appeared relatively resilient despite reduced discretionary space.

**Discussion:**

The findings reveal how administrative embedding shapes attitudinal professionalism in China's social service organizations. Methodologically, the study contributes a multidimensional operationalization of attitudinal professionalism; conceptually, it situates micro-level orientations within a layered framework spanning macro policy environments, meso organizational governance, and individual socialization. Implications for social work education, policy design, and organizational management are highlighted.

## Introduction

Although professionalization has long been a core concern in social work, recent decades of dramatic social, economic, and political changes have heightened the demand for professional social work services more than ever (NordesjÃű, 2020; Weiss-Gal and Welbourne, 2008). In response, the scholarly focus has increasingly shifted toward the role of individual practitioners and their professionalism as the proximate mechanism through which professional knowledge is enacted in practice (Bair, 2016; Evetts, 2003). Clarifying what we mean by professionalism and how it differs from professionalization is therefore essential for both theory and practice.

Conceptually, professionalization refers to the macro-level, historical process by which an occupation seeks to attain and maintain the status of a profession (Ritzer, 1975; Vollmer and Mills, 1966). Professionalism, by contrast, is a multi-level construct that can be understood through at least four distinct yet interconnected lenses: (1) a macro-level ideology that legitimizes professional authority and facilitates market closure (Johnson, 2015; Evetts, 2003); (2) an occupational value system comprising shared norms, ethics, and a commitment to public service (Evetts, 2013; Freidson, 1988); (3) a structural/organizational reality involving jurisdictional control, credentialing, and the influence of managerial regimes (Abbott, 1988; Evetts, 2011); (4) an individual-level attribute concerning the attitudes, values, and behaviors of practitioners themselves (Chen et al., 2019; Swailes, 2003). This study focuses specifically on attitudinal professionalism, situating it analytically within meso-level organizational embedding and macro-level policy regimes. Such an approach clarifies the layered framework connecting micro-level orientations to the governance arrangements and institutional logics that shape them.

Attitudinal professionalism refers to the set of individual values, behavioral orientations, and belief systems that shape how practitioners engage with their work (Hall, 1968; Lee, 2014). Social work professionalism produces welfare and tries to increase social justice (Heite, 2012). Grounded in the theory of planned behavior, which posits that attitudes shape intentions and actions (Ajzen, 1991), this construct reflects practitioners' propensity to act in ways that promote client wellbeing and service quality. Prior studies link stronger professional commitment, a core component of attitudinal professionalism, with higher performance and extra effort, and reduced withdrawal behaviors (Avolio et al., 2004; Mor Barak et al., 2001). Identifying factors associated with social workers' attitudinal professionalism can thus inform targeted interventions and workforce development strategies.

An attitudinal focus is particularly pertinent in China, given the diversity of its social work practitioners and the state-led, administratively embedded nature of the profession (Cai, 2021; Wang, 2011). Since the government's 2006 strategy to build a “great team of professional social workers,” the workforce has grown rapidly from about 200,000 in 2012 to 1.45 million in 2020 (Lei and Huang, 2018; Lei et al., 2021). This policy-driven expansion has resulted in considerable heterogeneity in educational backgrounds, certification pathways, and practice experiences. Many enter through an open licensure examination with modest practical requirements, while others serve as “street-level bureaucrats” without formal social work education, relying instead on localized competencies acquired in administrative or community roles (Song et al., 2023; Tang, 2018). Such diverse entry routes likely contribute to significant variation in professional identification and commitment, that is, in attitudinal professionalism (Wynd, 2003). At the same time, government's purchase-of-service arrangements have institutionalized standardized procedures, output-oriented evaluation, and managerial oversight as core government tools (Lei et al., 2022; Yin, 2011). These mechanisms embed agencies and practitioners within administrative and managerial logics that prioritize technical competence and measurable outcomes, often characterized as “pragmatic professionalism” (Lei and Huang, 2018), thereby influencing not only external practices but also internal value orientations. In sum, China's context combines diverse professional socialization pathways with strong administrative embedding, creating conditions under which attitudinal orientations may vary considerably and respond differentially to organizational and policy influences. Yet, how individual practitioners cultivate, sustain, or adapt their professionalism under these structural and ideological forces remains inadequately understood.

Against this backdrop, we investigate the current status of social workers' attitudinal professionalism and its associated factors in mainland China, drawing on data from Guangzhou, Shenzhen, and Shanghai. By explicitly situating micro-level attitudes within China's administratively embedded service regime, we clarify which facet of professionalism we measure, why an attitudinal lens is theoretically and practically informative, and how meso- and macro-level contexts may influence individual orientations. Our analysis contributes conceptually by operationalizing a layered (micro-meso-macro) framework of professionalism, empirically by providing multi-city evidence on attitudinal professionalism under state-led expansion and managerial governance, and practically by identifying organizational and policy-proximal levers that could strengthen professional commitment and enhance service quality.

## Literature review and conceptual framework

### Professionalism as a layered construct: from demarcation to levels

Classic scholarship on professions first attended to demarcation, which draws conceptual and institutional boundaries between professions and other occupations, before turning to explanations of how occupations attain professional standing (Abbott, 1988; Fournier, 1999). Within this trajectory, two influential strands took shape. Trait approach pursued definitional precision by specifying attributes such as a systematic knowledge base, formal training, and codified ethics (Carr-Saunders and Wilson, 1934; Millerson, 1964), whereas power approach emphasized how professions consolidate status through jurisdictional claims and market closure (Freidson, 1984; Larson, 1977).

Contemporary research extends these traditions by conceptualizing professionalism as a multidimensional, layered construct operating across macro, meso, and micro levels. At the macro level, professionalism functions as an ideology and discourse that legitimates occupational authority and sustains public trust (Davies, 1996; Johnson, 2015). It also encompasses an occupational value system stressing ethics, altruism, and service orientation as normative foundations of professional communities (Durkheim, 1992; Marshall, 2006; Parsons, 1951). At the meso level, professionalism is institutionalized through organizational and regulatory arrangements, including jurisdictional settlements, credentialing systems, and authority structures ranging from collegial to managerial (Evetts, 2011, 2013; Fenton, 2016). At the micro level, professionalism is enacted through practitioners' value commitments, beliefs, and role orientations in everyday practice (Hall, 1968; Snizek, 1972; Wynd, 2003). Adopting this layered view helps prevent conceptual conflation across levels and clarifies that micro-level orientations, while shaped by macro discourses and meso institutions, remain analytically distinct. This study focuses on the micro facet, attitudinal professionalism, while situating it within relevant organizational and policy contexts.

The concept of professionalism in social work, in recent years, has undergone renewed scrutiny, as scholars have sought to reconcile traditional value-based notions of the profession with managerial, neoliberal, and post-pandemic realities (Hugman, 2003). Tensions have long been existed in social work between its altruistic, value-driven mission and the managerial, efficiency-oriented contexts (Weiss-Gal and Welbourne, 2008). At the macro-level, social work professionalism is shaped by global discourses of human rights and social justice (Heite, 2012), which is often negotiated within distinct national welfare regimes and state-profession relationships. At the meso level, studies highlight how new public management reforms and output-based contracting, prevalent in many countries, reconfigure social work's institutional logics, often privileging managerial control over traditional collegial control (Tsui and Cheung, 2004; Willner and Heller, 2024). At the micro level, research examines how frontline social workers navigate these competing demands, striving to maintain a professional identity rooted in ethical commitment and client-centered practice despite organizational constraints (Andreassen and Natland, 2022; Liu et al., 2022). This body of work affirms the utility of a level-of-analysis approach for dissecting the complex and often contested nature of professionalism in social work.

### Attitudinal professionalism: definition and measurement

The study examines attitudinal professionalism, which is understood as the constellation of individual-level values, beliefs, and role orientations that underwrite professional conduct and sustain a sense of vocation (Hammer, 2000; Parkan, 2008). In line with Hall's (1968) professional model and subsequent operationalizations, we work with five interrelated components that together capture the micro facet in a theory-coherent manner, including professional organization reference, commitment to public service, endorsement of self-regulation, sense of calling, and perceived autonomy (e.g., Boyt et al., 2001; Chen et al., 2019; Wallace and Kay, 2008). In our usage, autonomy refers to experienced discretion in day-to-day practice rather than collective or structural autonomy rooted in jurisdictional control, credentialing, or governance. This distinction is important because it prevents institutional “facts” from being conflated with attitudinal responses. The attitudinal lens is not merely definitional. Following the theory of planned behavior, attitudes inform intentions and guide subsequent behavior (Ajzen, 1991). Empirically, research in human-service settings demonstrates that practitioners with higher identification with and commitment to the profession, which are core aspects of attitudinal professionalism, show higher performance and persistence, and lower absence and withdrawal (Avolio et al., 2004; Mor Barak et al., 2001; Wynd, 2003).

Each of the five components provides a micro-channel through which macro ideologies and meso institutions can be interiorized. Professional organization reference indexes identification with the occupational community and its normative jurisdiction and resonates with the collegial authority emphasized in occupational professionalism (Abbott, 1988; Evetts, 2013). Commitment to public service expresses the service ethic that undergirds legitimacy and public trust, thereby connecting individual commitment to the macro discourse of professional authority (Greenwood, 1957). Endorsement of self-regulation reflects a belief in peer-based quality assurance and thus mirrors, at an individual level, meso-level collective self-regulatory arrangements (Hall, 1968). Sense of calling captures the vocational and moral motivation associated with the professional logic, which Freidson (1984) contrasted with market and managerial logics. Finally, perceived autonomy registers the practitioner's discretionary space as experienced in practice and is the dimension most directly exposed to managerial or bureaucratic encroachment (Freidson, 1984). Read together, these dimensions map onto identity referents, service ethic, authority basis, motivational anchor, and discretionary space. Accordingly, taken as a set, they provide a minimal but conceptually integrated coverage of the attitudinal facet. Building on layered accounts of professionalism, we posit that administratively embedded service regimes, exemplified by government purchase of services and managerial controls, may differentially imprint these attitudinal facets (Freidson, 1984, 1999). This can produce a decoupling between value-oriented commitment (e.g., public service, calling, self-regulation) and discretion-related orientations (i.e., perceived autonomy), a pattern we term “value-discretion divergence.” We therefore adopt an explicitly multidimensional measurement strategy to permit a more nuanced, facet-level diagnosis of attitudinal professionalism.

### Professional socialization and organizational embedding in China

Variation in attitudinal professionalism can be coherently interpreted through the lens of professional socialization (Clouder, 2003). Socialization processes transmit knowledge, values, and role interactions, and organizational rules and routines (Barretti, 2004; Brim and Wheeler, 1966; Dinmohammadi et al., 2013; Miller, 2010). In this perspective, the covariates frequently used in prior work, such as gender, educational background, licensure or certification, and work experience, are not *ad hoc* controls but markers of differentiated socialization trajectories. Gendered pathways in human-service occupations can shape orientations toward service, calling, and identity persistence (Dominelli, 2017; Hall, 1968; Rumens and Kerfoot, 2009; Wuest, 1994). Formal education transmits professional knowledge and norms and may consolidate professional identity (Miller, 1988; Rutty, 1998; Tanaka et al., 2016). Licensure and certification signal credentialing, but in exam-centric systems they may be only weakly coupled to practice-based socialization, potentially yielding counterintuitive associations with attitudinal professionalism (Shi, 2021). Accumulated practice experience is often associated with heightened expertise, clearer role expectations, and stronger commitment (Keyton, 2011; Shafer et al., 2002; Suyono and Al Farooque, 2019; Yoder, 1995).

Organizational embedding further conditions these orientations. In China, social work organizations frequently operate under government purchase-of-service arrangements that institutionalize standardized procedures, output-focused evaluation, and managerial oversight (Feng and Ding, 2014; Peng, 2010; Wu, 2015). Evidence from adjacent professions suggests that managerialist environments can erode perceived discretion and reshape value priorities (Suddaby et al., 2009; Zheng et al., 2021). Under such regimes, value commitment linked to service, calling, and collegial self-regulation may persist even when perceived autonomy is constrained, generating the value-discretion divergence described above. Recognizing this possibility is important for interpreting attitudinal scores without imputing structural autonomy or jurisdictional control, and it reinforces the analytic payoff of a multidimensional attitudinal specification in the Chinese context.

A growing body of empirical research on Chinese social work provides concrete evidence for these dynamics. For instance, Li and Zeng (2022) suggested value-laden commitment promotes social workers' job satisfaction and prevents them from leaving. However, Liu and colleagues found in their study that social workers' attitudinal professionalism plays a role in preventing them from leaving, but the organization's managerial control diminishes that effect (Liu et al., 2022). Moreover, Zhang et al. (2022) demonstrated that professional discretion can coexist with bureaucratic dependence as long as more professional agents are involved, such as practitioners with social work educational background and professional organizations. Wu (2020) also found the coexistence of bureaucratic dependence and moral commitment within social service agencies. Accordingly, current literature has not fully addressed how professionalism is experienced within the uniquely embedded organizational and policy context of Chinese social service agencies (Lei et al., 2022).

### Conceptual framework and associational expectations

**Figure 1:**
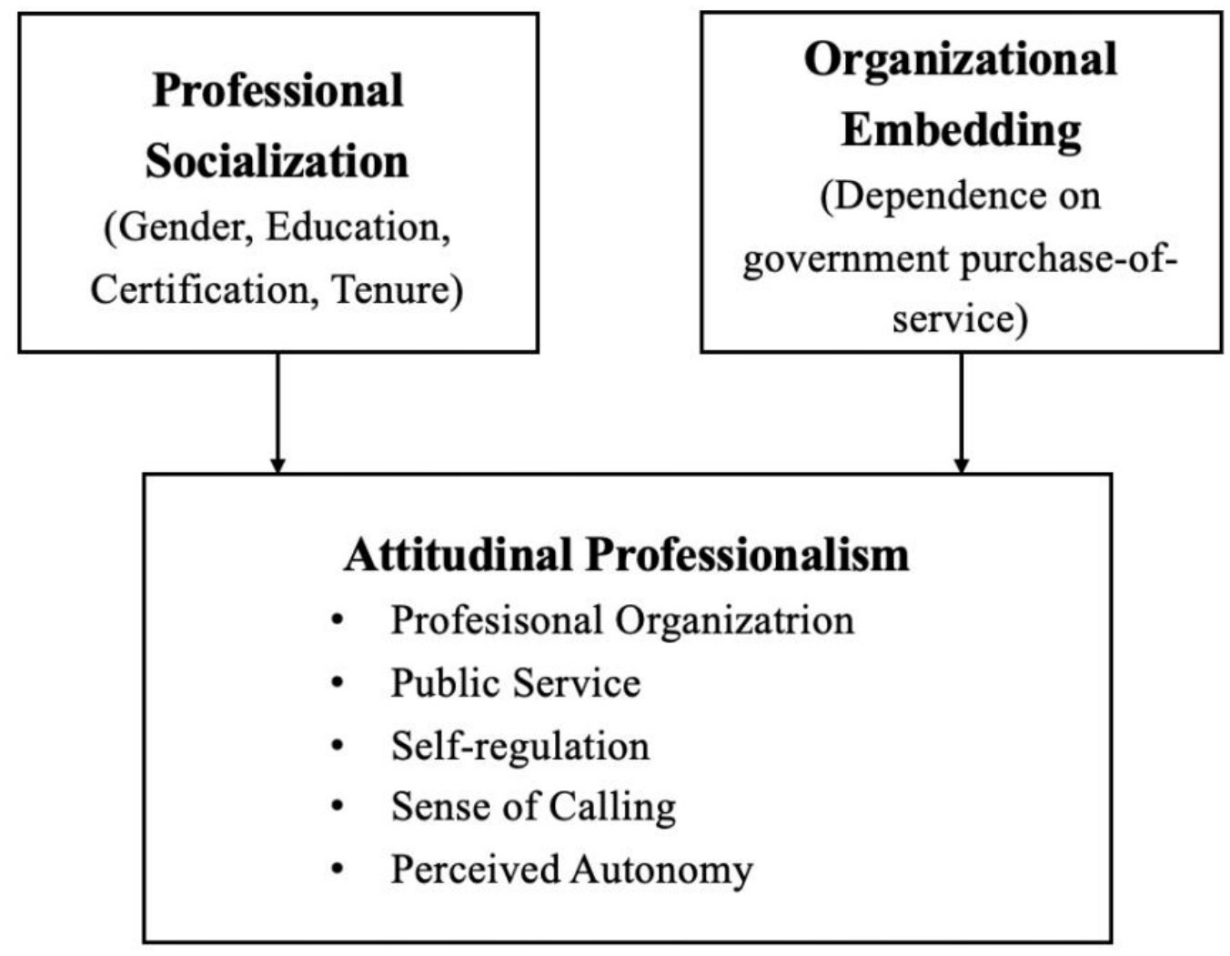
Conceptual framework linking socialization, organizational embedding, and attitudinal professionalism among Chinese social workers.

Integrating these strands, we conceptualize attitudinal professionalism, as operationalized by the five components derived from Hall's model, as an individual-level outcome shaped by professional socialization routes and organizational embedding (see Figure 1). Socialization is captured through gendered pathways, education, licensure, and tenure, which function as observable traces of how values and role expectations have been transmitted and internalized. Organizational embedding is operationalized as organizational dependence on government purchase of services, a salient feature of China's administratively embedded regime that is directly observable in our data. This formulation is consistent with the layered view of professionalism while remaining empirically tractable.

Within this framework, we expect organizations with higher dependence on government purchase of services to exhibit lower perceived autonomy, reflecting constraints on discretionary space characteristic of administratively embedded agencies, with comparatively weaker associations for value-oriented facets such as public service and calling (Tummers and Bekkers, 2014; Wang, 2011). Along socialization pathways, longer tenure is anticipated to be positively associated with the internalization of norms linked to self-regulation and calling, while the direction and magnitude of associations for education and licensure are treated as empirical questions given the exam-centric and heterogeneous entry routes that may attenuate their coupling with practice-based socialization. The empirical strategy, accordingly, employs a multidimensional specification to detect whether and how attitudinal facets covary or diverge across organizational embedding and socialization trajectories.

## Methods

### Sampling and participants

We purposively selected Guangzhou, Shenzhen, and Shanghai as field sites for three reasons. First, these coastal megacities are widely recognized as policy front-runners in the development of social work and the institutionalization of government purchase of services, providing a mature context in which attitudinal professionalism is salient and observable. Second, each city hosts a diverse ecology of agencies, from government-dependent organizations to more independent, community-based providers, thus offering the organizational variation (especially in dependence on government purchase) required by our conceptual framework. Third, the cities maintain comprehensive, publicly accessible rosters of social work organizations, which enabled a transparent multi-stage sampling design.

A multi-stage cluster sampling design was then implemented. In each city, five districts were randomly selected. Lists of social work organizations were compiled from the official municipal portals. To ensure organizational stability and relevance to frontline practice, inclusion criteria were: (1) founded for at least 1 year; (2) providing direct services; and (3) having at least 10 full-time employees. To preserve heterogeneity by scale, organizational rosters were stratified by size: agencies with more than 30 full-time social workers were classified as medium-to-large, and those with fewer than 30 full-time social workers were considered small organizations. From each sampled district, two organizations (one medium-to-large and one small) were randomly selected, yielding 30 organizations identified across the three cities. Invitations were sent by email or telephone. Ultimately, 7/10 organizations in Guangzhou and 8/10 in Shenzhen consented and participated; in Shanghai, 2/10 organizations agreed to participate. Employees from the consenting organizations constituted the study sample. Non-response at the organizational level (17/30 participating) and the focus on three developed metropolitans constrain external validity. Therefore, findings should be generalized primarily to similar urban contexts rather than to inland or county-level settings.

**Figure 2:**
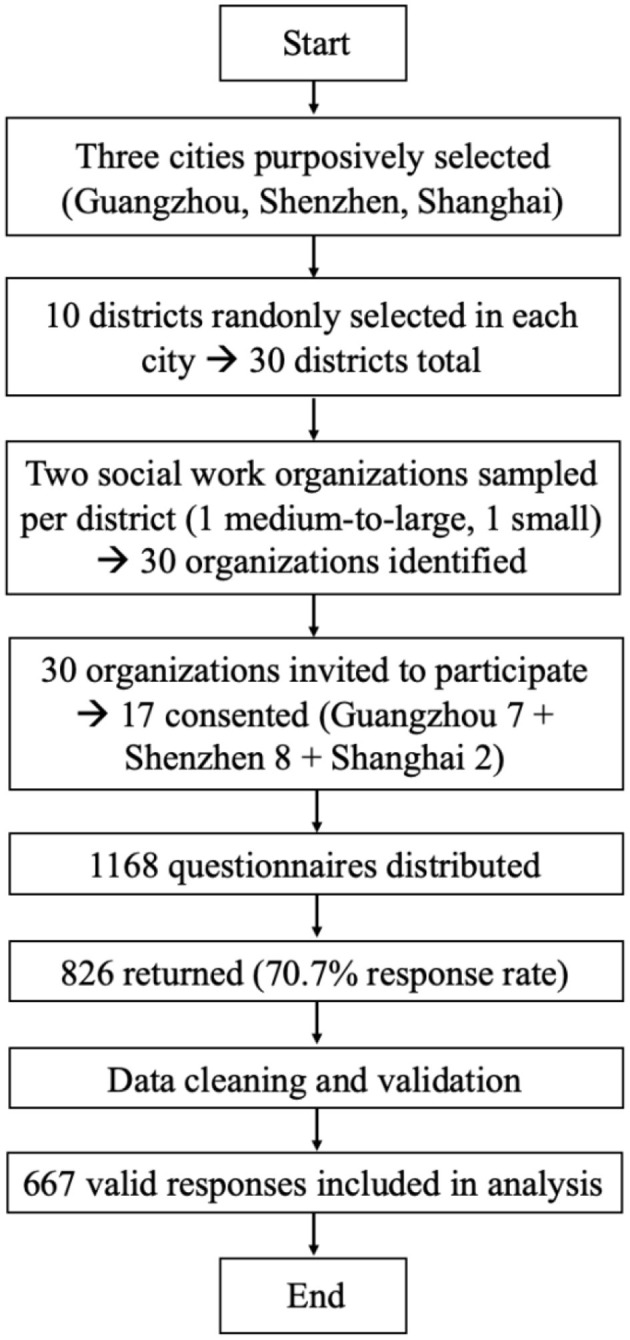
Flowchart of multistage sampling and survey response process among Chinese social workers.

Data were collected via an online questionnaire administered between October 2021 and January 2022. Each organization distributed the survey link internally, and respondents completed the instrument independently. Informed consent was obtained at the start of the survey. Participation was voluntary and anonymous, and respondents were informed that data would be used solely for academic purposes. In total, 1,168 questionnaires were distributed and 826 were returned, a 70.7% response rate. After excluding invalid responses, 667 questionnaires were retained for analysis. Figure 2 illustrates the multistage sampling and survey response process. In addition, Appendix 1 explains the data cleaning procedure in detail.

### Measurements

#### Professionalism

Professionalism was measured using Hall's (1968) Professionalism Inventory Scale. Snizek (1972) modified Hall's scale to create a shortened version named the Snizek-revised Hall's Professionalism Inventory Scale (SR-HPIS). In this study, professionalism was measured by 16 items on five dimensions, adapted from Snizek's (1972) shortened version of Hall's professionalism scale. The scale had previously been validated in the Chinese context among clinical nurses (Chen et al., 2019), and was thus already translated into Chinese. The dimension “Use of professional organizations” (the Professional Organization subscale) included four items (e.g., I systematically read the professional journals); “Belief in public service” (the Public Service subscale) included three items (e.g., if ever an occupation is indispensable, it is social work); “Belief in self-regulation” (the Self-regulation subscale) contained three items (e.g., My fellow professionals have a pretty good idea about each other's competence); “Sense of calling to the field” (the Sense of calling subscale) contained four items (e.g., Social workers have a real sense of “calling” to their work); and “Perceived autonomy” (the Perceived Autonomy subscale) included two items (e.g., I make my own decisions in regard to what is to be done in my work). All items were assessed on a 7-point Likert scale ranging from “1 = totally disagree” to “7 = totally agree.” The subscale scores were computed as mean scores, with higher scores indicating more positive attitudes toward professionalism. The reliability of the scale used in the present study was 0.74.

#### Organization independence

In China, social work agencies may be established by government bodies or registered as private non-governmental organizations. Regardless of legal origin, many agencies rely heavily on municipal “purchase-of-service” contract as their main source of operating revenue. Because financial dependence typically entails administrative dependence, often through standardized procedures, output-oriented evaluation, and multilayered reporting, we used organizational dependence on government purchase-of-service contracts as an observable indicator of organizational embedding (Gazley, 2008; Smith and Lipsky, 2009; Young, 2000). To capture financially induced administrative embedding, agencies were dichotomized according to whether government purchase-of-service contracts constituted a majority of their operating revenue in the preceding fiscal year. Agencies meeting this criterion were coded as dependent (1), and those deriving less than a majority of their revenue from such contracts were coded as independent (0). Revenue proportions were reported by agency liaisons and, when available, verified against internal financial summaries.

#### Professional socialization indicators

Following the study's conceptual framing, four individual-level variables, including gender, years of work experience, educational level, and qualification certification, are treated as indicators of differentiated professional socialization trajectories. Each reflects distinct entry routes and formative experiences that shape how practitioners internalize and enact professional values. First, gender was coded as a binary variable (0 = female; 1= male). Second, years of work experience was coded as an ordered categorical variable with four levels: 1 = 0–2 years, 2 = 3–5 years, 3 = 6–10 years, and 4 = more than 10 years. Third, educational level was coded as 1 = secondary/vocational or below, 2 = junior college, 3 = bachelor's degree, and 4 = master's degree or above. Fourth, qualification certification was coded as 1 = holds a social work qualification or licensure, 0 = does not hold such certification.

#### Data analysis

First of all, descriptive statistics (frequencies, percentages, means and standard deviations) for the variables involved in the study were conducted. In addition, multiple regression analysis was performed to examine the association of the variables with social workers' professionalism. Specifically, six multiple regression models were conducted, using the total SR-HPIS scale and its five subscales as dependent variables, respectively. Given the evidence form previous studies, years of experience in the field of social work, educational level, certification, and organization independence were included in the regression model. A 0.05 level of significance was established for all hypothesis testing. All analyses were carried out using the SPSS 24.0 software.

## Results

### Preliminary analyses

Tables 1–3 present descriptive statistics of study variables (*N* = 667). A large proportion of the sample was female (80.8%). More than half of the participants had a bachelor's degree or above (56.1%), and had certain type of social work qualification certification (65.5%). In terms of years of work experience in the field of social work, 42.3% had been in the field for 2–5 years, followed by 34.3% for less than 2 years, 17.3% in the field for 6–10 years, and 6.1% for more than 10 years. The vast majority of the sample worked for organizations that were heavily dependent on government's purchase of social services for living (90%).

**Table 1:** Descriptive statistics of sociodemographic characteristics of sample social workers, Guangzhou, Shenzhen, and Shanghai, China, 2021–2022 (N = 667).

**Characteristics**	**Frequency (*N*)**	**Percentage (%)**
**Gender**		
Male	128	19.2
Female	539	80.8
**Education**		
HS/TS diploma	31	4.6
JC/VC degree	262	39.3
Bachelor's degree or above	374	56.1
**Certificate**		
Certification	437	65.5
Non-certification	230	34.5
**Years of experience**		
< 2 years	229	34.3
2–5 years	282	42.3
6–10 years	115	17.2
10+ years	41	6.1
**Funding**		
Government purchase	600	90.0
Others	67	10.0

HS, high school; TS, technical secondary school; JC, junior college; VC, vocational college.

**Table 2:** Descriptive statistics on total SR-HPIS and five subscales' scores of sample social workers, Guangzhou, Shenzhen, and Shanghai, China, 2021–2022 (*N* = 667).

	**Mean**	**SD**
SR-HPIS	4.39	0.79
**Subscales**		
Professional organization	4.75	1.15
Public service	4.17	1.09
Self-regulation	4.51	0.99
Sense of calling	4.88	1.21
Perceived autonomy	3.65	1.19

SR-HPIS, Snizek-revised Hall's Professionalism Inventory Scale.

**Table 3:** Means and standard deviations (SDs) of each scale by demographic characteristics groups of sample social workers, Guangzhou, Shenzhen, and Shanghai, China, 2021–2022 (SDs in parenthesis).

	**Gender**		**Education**			**Certification**		**Years of experience**				**Organization independence**	
	**Male (*****n*** = **128)**	**Female (*****n*** = **539)**	**HS/TS (*****n*** = **31)**	**JC/VC (*****n*** = **262)**	**BA or above (*****n*** = **374)**	**Yes (*****n*** = **437)**	**No (*****n*** = **230)**	<**2 (*****n*** = **229)**	**2–5 (*****n*** = **282)**	**6–10 (*****n*** = **115)**	>**10 (*****n*** = **41)**	**GP (*****n*** = **600)**	**Others (*****n*** = **67)**
Total SR-HPIS	4.41 (0.82)	4.39 (0.78)	4.70 (0.81)	4.55 (0.81)	4.26 (0.74)	4.32 (0.76)	4.53 (0.83)	4.38 (0.78)	4.38 (0.79)	4.36 (0.75)	4.61 (0.92)	4.34 (0.77)	4.84 (0.82)
**Subscales**													
PO	4.82 (1.30)	4.73 (1.12)	5.00 (1.15)	4.94 (1.09)	4.60 (1.18)	4.68 (1.14)	4.87 (1.17)	4.76 (1.12)	4.74 (1.13)	4.71 (1.18)	4.84 (1.49)	4.70 (1.14)	5.16 (1.21)
PS	4.08 (1.14)	4.19 (1.07)	4.47 (1.07)	4.38 (1.06)	4.00 (1.08)	4.07 (1.06)	4.36 (1.11)	4.15 (1.06)	4.12 (1.10)	4.24 (1.05)	4.42 (1.23)	4.11 (1.07)	4.71 (1.07)
SR	4.47 (1.08)	4.52 (0.96)	4.73 (0.94)	4.69 (0.97)	4.37 (0.99)	4.44(0.97)	4.66 (1.00)	4.50 (0.94)	4.55 (1.02)	4.39 (0.94)	4.70 (1.15)	4.48 (0.96)	4.82 (1.15)
SC	4.90 (1.19)	4.87 (1.22)	5.23 (1.22)	5.05 (1.23)	4.72 (1.18)	4.77 (1.20)	5.08 (1.21)	4.95 (1.18)	4.79 (1.23)	4.80 (1.22)	5.29 (1.23)	4.80 (1.21)	5.53 (1.06)
AU	3.80 (1.28)	3.61 (1.16)	4.06 (1.24)	3.69 (1.12)	3.59 (1.19)	3.63 (1.17)	3.69 (1.21)	3.54 (1.17)	3.72 (1.23)	3.68 (1.12)	3.78 (1.14)	3.62 (1.17)	3.99 (1.26)

SR-HPIS, Snizek-revised Hall's Professionalism Inventory Scale; HS, high school; TS, technical secondary school; JC, junior college; VC, vocational college; BA, Bachelor's degree; GP, government purchase; PO, professional organization; PS, public service; SR, self-regulation; SC, sense of calling; AU, perceived autonomy.

The mean composite professionalism score achieved by the 667 social workers was 4.39 (*SD* = 0.79). In term of subscales, the highest score was obtained in Sense of Calling (M = 4.88, *SD* = 1.21), while the lowest score was Perceived Autonomy (M = 3.65, *SD* = 1.19). Male social workers (M = 4.41, *SD* = 0.82) had higher total professionalism score than females (M = 4.39, *SD* = 0.78). High school or technical secondary school graduated social workers had the highest score on total professionalism (M = 4.70, *SD* = 0.81), followed by junior or vocational college graduates (M = 4.55, *SD* = 0.81), and then the individuals holding a bachelor's degree or above (M = 4.26, *SD* = 0.74). Social workers with qualification certification (M = 4.32, *SD* = 0.76) scored lower on total professionalism than their counterparts not having any certifications (M = 4.53, *SD* = 0.83). The most experienced social workers (more than 10 years of experience) scored the highest on total professionalism (M = 4.61, *SD* = 0.92), while social workers have been in the field between 6 and 10 years scored the lowest (M = 4.36, *SD* = 0.75). Moreover, social workers working in independent organization (not primarily relying on government purchase) scored higher on total professionalism (M = 4.84, *SD* = 0.82) than their counterparts in dependent organizations (M = 4.34, *SD* = 0.77).

In order to examine multi-collinearity among independent variables and covariates, the variance inflation factor (VIF) test was conducted, and VIF scores ranged from 1.080 to 1.257. Considering that VIF scores were all much less than 10, nearly no problematic multicollinearity existed (Hair et al., 2010). Moreover, residual normality was inspected using Q-Q plots and found to be acceptable given the large sample size. In addition, there was independence of residual, as assessed by a Durbin–Watson statistic of 1.900.

### Regression results

Tables 4, 5 show findings from the multiple regression models using the five professionalism subscales and the total SR-HPIS scale as dependent variables, respectively. Male and female social workers had no significant differences in total professionalism scores as well as five attitudinal attributes' scores. Having qualification certification was negatively associated with the subscale of Public Service [β = −0.243, 95% CI (−0.438, −0.047)], indicating that practitioners who had not been identified as qualified social workers believed in that his/her profession is both indispensable and beneficial to society more than did their counterparts who had qualification certifications. Accordingly, social workers not having certification had higher levels of professionalism, as reflected in the association of certification and the total SR-HPIS scale [β = −0.156, 95% CI (−0.297, −0.014)]. In addition, organization independence was positively associated with the total SR-HPIS scale [β = 0.436, 95% CI (0.230, 0.642)], as well as four subscales, including Professional Organization [β = 0.401, 95% CI (0.092, 0.711)], Public Service [β = 0.565, 95% CI (0.280, 0.850)], Self-Regulation [β = 0.286, 95% CI (0.022, 0.549)], and Sense of Calling [β = 0.633, 95% CI (0.312, 0.954)]. This indicates that social workers working in independent organizations had higher levels of professionalism, and were more conscious about using professional organizations as major referents, believing more in public service, as well as self-regulation, and more devoted to his/her work, comparing to social workers in dependent organizations. However, independence had non-significant association with the scale of perceived autonomy. Social workers graduated from junior or vocational college had higher scores in Professional Organization [β = 0.317, 95% CI (0.130, 0.505)], Public Service [β = 0.356, 95% CI (0.183, 0.529)], Self-regulation, and Sense of Calling [β = 0.282, 95% CI (0.123, 0.442)] than those who had a bachelor's degree or above (i.e., the reference group). Moreover, social workers holding a junior college or vocational college's degree had global attitudes toward the profession of social work, which were more positive than the attitudes of those who had a Bachelor's degree or above [β = 0.270, 95% CI (0.145, 0.395)]. Respondents with less than 2 years of work experience [β = −0.442, 95% CI (−0.848, −0.037)] and 2–5 years of experience [β = −0.474, 95% CI (−0.867, −0.081)] in the field of social work felt less calling from the field than their counterparts in the field for more than 10 years (i.e., the reference group). Further, social workers with less than 2 years of experience possess a weaker belief in Public Service [β = −0.407, 95% CI (−0.767, −0.047)] than those more experienced counterparts (more than 10 years of experience). In general, there was relatively strong evidence showing that years of experience was negatively associated with social workers' professionalism, specifically indicating that social workers with less than 2 years of experience had lower levels of professionalism than those who had been in the field for more than 10 years [β = −0.313, 95% CI (−0.573, −0.052)].

**Table 4:** Regression results with the five SR-HPIS subscales (data collected from sample social workers in Guangzhou, Shenzhen, Shanghai, China, 2021–2022, *N* = 667).

	**Professional organization**					**Public service**				
	***β***	**SE**	***B***	**95% CI**		***β***	**SE**	***B***	**95% CI**	
Intercept	4.786^***^	0.225	0.000	4.345	5.227	4.261^***^	0.207	0.000	3.855	4.667
Gender (ref: male)	−0.051	0.113	−0.017	−0.274	0.171	0.178	0.104	0.065	−0.027	0.383
Certification (ref: no certification)	−0.104	0.108	−0.043	−0.317	0.108	−0.243^*^	0.100	−0.106	−0.438	−0.047
Funding (ref: government purchase)	0.401^*^	0.158	0.105	0.092	0.711	0.565^***^	0.145	0.157	0.280	0.850
**Education (ref: Bachelor's or above)**										
High school/technical secondary school	0.173	0.228	0.032	−0.274	0.620	0.109	0.210	0.021	−0.302	0.521
Junior college/vocational college	0.317^**^	0.096	0.134	0.130	0.505	0.356^***^	0.088	0.160	0.183	0.529
**Years of work experience (ref:** >**10 years)**										
< 2 years	−0.155	0.199	−0.064	−0.546	0.237	−0.407^*^	0.183	−0.178	−0.767	−0.047
2–5 years	−0.093	0.193	−0.040	−0.472	0.286	−0.298	0.178	−0.136	−0.647	0.050
6–10 years	−0.049	0.209	−0.016	−0.459	0.361	−0.072	0.192	−0.025	−0.450	0.305
*R* ^2^	0.035					0.075				
	**Self-regulation**					**Sense of calling**				
	β	**SE**	***B***	**95% CI**		β	**SE**	***B***	**95% CI**	
Intercept	4.629^***^	0.191	0.000	4.253	5.005	5.199^***^	0.233	0.000	4.741	5.656
Gender (ref: male)	0.082	0.097	0.033	−0.108	0.271	0.037	0.118	0.012	−0.194	0.267
Certifacation (ref: no certification)	−0.174	0.092	−0.084	−0.355	0.007	−0.181	0.112	−0.071	−0.402	0.039
Funding (ref: government purchase)	0.286^*^	0.134	0.087	0.022	0.549	0.633^***^	0.164	0.157	0.312	0.954
**Education (ref: Bachelor's or above)**										
High school/technical secondary school	0.153	0.194	0.033	−0.228	0.534	0.107	0.236	0.019	−0.357	0.571
Junior college/vocational college	0.282^**^	0.081	0.140	0.123	0.442	0.297^**^	0.099	0.120	0.102	0.492
**Years of work experience (ref:** > **10 years)**										
< 2 years	−0.301	0.170	−0.145	−0.634	0.032	−0.442^*^	0.207	−0.173	−0.848	−0.037
2–5 years	−0.158	0.164	−0.079	−0.481	0.165	−0.474^*^	0.200	−0.193	−0.867	−0.081
6–10 years	−0.247	0.178	−0.094	−0.596	0.103	−0.386	0.217	−0.120	−0.812	0.039
*R* ^2^	0.045					0.060				
		**Perceived autonomy**								
		β		**SE**		***B***	**95% CI**			
Intercept		3.844^***^		0.232		0.000	3.389		4.300	
Gender (ref: male)		−0.154		0.117		−0.051	−0.384		0.076	
Certifacation (ref: no certification)		−0.076		0.112		−0.031	−0.296		0.143	
Funding (ref: government purchase)		0.295		0.163		0.075	−0.025		0.614	
**Education (ref: Bachelor's or above)**					
High school/technical secondary school		0.322		0.235		0.057	−0.139		0.784	
Junior college/vocational college		0.097		0.099		0.040	−0.097		0.291	
**Years of work experience (ref:** >**10 years)**					
< 2 years		−0.259		0.206		−0.104	−0.663		0.145	
2–5 years		−0.012		0.199		−0.005	−0.404		0.379	
6–10 years		−0.034		0.216		−0.011	−0.458		0.389	
*R* ^2^		0.023								

SR-HPIS, Snizek-revised Hall's Professionalism Inventory Scale; β, standardized regression coefficient; B, unstandardized regression coefficient; SE, standard error; CI, confidence interval.

^*^p < 0.05; ^**^p < 0.01; ^***^p < 0.001.

**Table 5:** Regression results with the total SR-HPIS (data collected from sample social workers in Guangzhou, Shenzhen, Shanghai, China, 2021–2022, *N* = 667).

	**Total SR-HPIS**				
	β	**SE**	***B***	**95% CI**	
Intercept	4.544^***^	0.150	0.000	4.250	4.838
Gender (ref: male)	0.018	0.075	0.009	−0.130	0.166
Certification (ref: no certification)	−0.156^*^	0.072	−0.094	−0.297	−0.014
Funding (ref: government purchase)	0.436^***^	0.105	0.167	0.230	0.642
**Education (ref: Bachelor's or above)**					
High school/technical secondary school	0.173	0.152	0.064	−0.125	0.471
Junior college/vocational college	0.270^***^	0.064	0.168	0.145	0.395
**Years of work experience (ref:** >**10 years)**					
< 2 years	−0.313^*^	0.133	−0.189	−0.573	−0.052
2–5 years	−0.207	0.129	−0.130	−0.459	0.045
6–10 years	−0.158	0.139	−0.076	−0.431	0.115
*R* ^2^	0.080				

SR-HPIS, Snizek-revised Hall's Professionalism Inventory Scale; SE, standard error; CI, confidence interval.

^*^p < 0.05; ^***^p < 0.001.

## Discussion

### Main pattern and profile-level interpretation

Viewing professionalism as a multidimensional attitudinal construct reveals a consistent profile that would be obscured by a composite score. Respondents across cities reported stronger endorsement of public service and sense of calling, alongside lower perceived autonomy. Organizational independence, defined as lower reliance on government purchase-of-service, was positively associated with value-oriented facets (professional organization reference, public service, self-regulation, and calling), but showed a weak or non-significant link with perceived autonomy. Shorter tenure correlated negatively with sense of calling and the overall professionalism score; certification was negatively associated with public service and the composite measure; and lower formal education corresponded to higher scores on several subscales. These patterns reflect associational trends in professional dispositions, rather than structural or jurisdictional attributes.

Viewed at the profile level, the results indicate a value-discretion divergence. Specifically, robust value commitments coexist with comparatively compressed perceptions of discretionary space. This configuration is consistent with layered accounts that differentiate occupational professionalism, grounded in collegial norms and a public-regarding ethic, from organizational professionalism, characterized by standardization, output metrics, and managerial oversight (Evetts, 2003, 2013; Freidson, 1984). On this reading, managerial controls and audit logics register most immediately on perceived discretion, while value commitment that is more slowly socialized and normatively anchored exhibit greater resilience. Crucially, the divergence becomes visible precisely because professionalism is specified multidimensionally at the micro level. A single composite score would have masked the distinct sensitivity of discretion-related attitudes and overlooked the coexistence of strong value commitment and low perceived autonomy. Therefore, by clarifying that our focus is on attitudinal professionalism rather than structural power we demonstrate the theoretical and diagnostic value of a facet-level analysis.

Two interpretive notes are warranted before turning to mechanisms. First, the models show modest explanatory power (e.g., *R*^2^ =0.08 for the composite index), which is common in studies of complex attitudinal outcomes in heterogeneous service contexts (Aiken and West, 1991; Cohen et al., 2013; Falk and Heckman, 2009). The goal of the analysis is to recover patterned association rather than to achieve high prediction accuracy. Therefore, the findings should be read as significant but modest associations with practical implications at organizational touchpoints (e.g., supervision design, contracting requirements).

### Socialization, credentialing, and organizational embedding

A professional socialization lens renders the component-specific associations plausible and informative. The negative relationship between shorter tenure and sense of calling (and the composite) aligns with the view that practice-based socialization and role clarity accumulate over time, consolidating vocational identity and commitment (Suyono and Al Farooque, 2019; Tanaka et al., 2016; Wynd, 2003; Yoder, 1995). The counterintuitive links where lower education corresponds to higher scores on several facets and certification is negatively associated with public service and the composite corresponds can be understood within China's exam-focused credentialing system (Shi, 2021). Assessment systems foregrounding theoretical mastery and written testing may be only weakly coupled to practice-embedded learning, thereby generating expectation-reality gaps when graduates enter output-focused, administratively embedded settings (Li et al., 2012; Wang et al., 2019). By contrast, trajectories marked by intensive field exposure, structured supervision, and peer learning are more likely to translate formal knowledge into durable attitudinal commitments and context-appropriate discretion (Wei, 2014; Zhu and Chen, 2013).

The organizational findings point to a selective imprint of embedding. Contexts correlated with greater independence often sustain discursive and collegial supports, such as professional referencing, peer consultation, and value-affirming supervision, that reinforce identification with the professional community, endorsement of peer-based quality assurance, and vocational commitment (Noordegraaf, 2015, 2020). Yet perceived discretionary space can remain constrained by city- or system-level administrative logics, in terms of standardized procedures, contracting requirements, external evaluation metrics, that operate above the individual agency. In short, organizational independence may be sufficient to cultivate the value core of attitudinal professionalism but insufficient to expand perceived autonomy where external managerial controls remain strong. Read in this way, the attitudinal lens is not merely descriptive, while it is practically informative because it isolates the micro-channels through which organizational routines and educational design shape internal orientations under managerial governance.

### Situating the findings in China's administratively embedded regime

The attitudinal profile revealed by the study that elevated scores on value-oriented facets alongside lower perceived autonomy becomes most intelligible when micro-level attitudes are situated within China's administratively embedded regime of social service delivery. Since the mid-2000s, municipal purchase-of-service instruments have institutionalized standardized procedures, short contract cycles, performance audits, and multilayer reporting systems, positioning social work agencies as quasi-public providers whose operations are tightly coupled to government oversight and evaluation (Xu and Li, 2013). Within this governance configuration, value-oriented commitments, including public service motivation, collegial self-regulation, and vocation-like calling, are affirmed and legitimated by public discourse, civic expectations, and mission-framed organizational narratives (Jiang and Wang, 2016; Xu, 2023). At the same time, experiential discretion—the perceived capacity to exercise judgment in day-to-day practice—is compressed by pre-set targets, procedural templates, and evaluation indicators that cascade through administrative hierarchies.

Diverse entry routes interact with this regime to produce uneven practice-integrated socialization. Exam-centric credentialing with modest practical thresholds, rapid workforce scaling, and the recruitment of street-level personnel without professional training have contributed to wide variation in professional formation (Hou and Nie, 2021; Zhu and Tong, 2017). These structural characteristics help explain why value-oriented facets appear comparatively robust, while perceived autonomy is more sensitive to organizational dependence on contract-based governance. In China's state-led expansion of social work, ideological alignment and service-oriented values are explicitly endorsed through government discourse, professional registration campaigns, and agency narratives that frame social work as a moral and civic vocation (He et al., 2021). Even for entrants without formal training, these values are readily internalized through organizational induction and performance rhetoric that foreground social responsibility and public service. By contrast, the sense of professional discretion, understood as experienced autonomy in making case-level judgments, is directly constrained by the mechanisms through which services are procured and evaluated. Agencies that rely heavily on purchase-of-service contracts face short funding cycles, detailed procedural requirements, and output-based audits that narrow workers' perceived room for judgment. As a result, moral and value commitment remain institutionally reinforced, whereas experiential discretion becomes contingent on the degree of organizational embedding within the contractual governance system.

The coexistence of affirmed values and constrained discretion thus represents not a paradox but a patterned outcome of how macro policy expansion and meso-level managerial governance jointly condition micro-level professional orientations. Framed in these terms, our findings substantiate the paper's conceptual premise in the Chinese context, that is, in administratively embedded urban systems, professionalism is best examined as an attitudinally embodied construct that is selectively conditioned by institutional embedding. In doing so, the analysis links individual orientations to organizational embedding and the wider policy architecture that organizes them, making their interdependence empirically visible.

### Integrative synthesis: layered linkage and study contributions

Overall, this study operationalizes a layered framework of professionalism and illustrates how macro- and meso-level conditions are reflected in micro-level attitudinal data. Value-related dimensions respond differently than discretion-related ones under administrative embedding, producing a value-discretion divergence that composite measures would conceal. At the meso level, reliance on government purchase-of-service correlates with lower perceived autonomy and weaker value facet scores, indicating that managerial oversight affects not only task structure but also professional identity, self-regulation, and motivation. At the macro-level, state-led workforce expansion and contract-based governance from the institutional backdrop for these micro-meso dynamics.

Conceptually, this study refines attitudinal professionalism as a structured, five-facet construct whose components exhibit asymmetric sensitivity to connect. Mapping the Hall's component onto a layered framework shows that public service, sense of calling, and self-regulation function as value-oriented channels that remain comparatively resilient under administrative embedding, whereas perceived autonomy operates as a discretion-oriented channel that is selectively conditioned by organizational embedding. In addition, professional organization reference bridges value and governance by linking identity to collegial authority. This yields a clear, testable claim that in administratively embedded regimes, value facets tend to decouple from discretion facets, with the latter more elastic to meso-level governance. The contribution lies not in enumerating dimensions but in specifying their relational positions within the layered architecture and clarifying their differential coupling to organizational logics, thereby explaining why composite indices can mask systematic divergence and generating falsifiable expectations for other institutional settings.

Empirically, the study draws on multi-city evidence from policy-leading urban systems to document patterned variation in attitudinal professionalism under state-led expansion and managerial governance. Treading the five facets directly, rather than collapsing them, proves diagnostically productive: only a facet-level specification reveals the value-discretion profile and makes the cross-level linkage empirically tractable. The analysis also identified policy-proximal levers by locating the socialization nodes (education, certification, tenure) and embedding features (purchase-of-service dependence) most closely associated with the value core and with perceived discretion. By situating micro-level attitudes within an administratively embedded regime, the study shows how meso- and macro-level conditions are reflected in individual orientations in contemporary Chinese social work, while setting out propositions that can be probed in contexts where collegial authority and organizational autonomy take different forms. Accordingly, we advance the following testable proposition: in administratively embedded service regime, the value-oriented facets of attitudinal professionalism, in terms of public-service commitment, sense of calling, and endorsement of peer self-regulation, tend to remain comparatively stable, whereas perceived autonomy is more elastic and more sensitive to organizational embedding.

## Implications

The observed value-discretion divergence indicates that organizations can strengthen attitudinal professionalism by enlarging perceived discretionary space in day-to-day practice without presupposing immediate, system-wide structural reforms. A layered reading suggests that micro-level dispositions are malleable at meso- and policy-proximal touchpoints. Within agencies structured reflective supervision, in terms of regular case reflection, peer case conferences, and supervisor coaching oriented to judgment rather than throughput, can legitimize the use of discretion in complex cases and anchor it in professional values (Guo and Guo, 2013). Clarifying roles and shaping workload design in ways that protect judgment-intensive work, such as manageable caseloads, scheduled time for reflection, explicit decision rights, further prevents administrative load from crowding out experiential discretion (Guo, 2014). On the contracting side, rebalancing metrics within purchase-of-service regimes to complement output counts with quality-of-practice indicators, such as supervision dosage, protected reflective time, and client-reported process quality, aligns performance management with the professional logic while preserving accountability. These adjustments do not alter structural or jurisdictional autonomy, but they expand the discretionary space practitioners perceive and use, sustaining the value core that remains comparatively robust in administratively embedded settings.

Patterns by education and certification point to a second lever: tightening the coupling between theoretical training and field practice so that value commitments and context-appropriate discretion are internalized together. Universities and training providers can deepen practice-integrated curricula through longer, better-supervised practicums, co-teaching by practitioners and academics, and reflective seminars that explicitly connect public-service ethics and calling to discretionary judgment under bureaucratic constraints. Structured debriefs that surface and address expectation-reality gaps can help early-career practitioners reconcile assessed competencies with the demands of output-focused regimes (Wang et al., 2019). Credentialing bodies can complement examinations with practice-based requirements and formative feedback, in terms of supervised practice hours, observed assessments, reflective portfolio, so that candidates experience discretion as a legitimate and assessable element of professional work (He et al., 2021). Agencies can then target onboarding and mentoring to bolster facets that appear comparatively lower (especially perceived discretion) while consolidating facets that are already strong (public service, calling).

Finally, purchasers and funders operating government purchase-of-service regimes have proximate tools to mitigate inadvertent compression of discretion. For instance, contract specifications can carve out protected reflective time, and scoring rubrics can help recognize supervision quality. Moreover, hybrid performance frameworks can pilot modest weights for client-reported relational and process quality. Because these design tweaks operate at the meso-policy interface they are well-suited to the administratively embedded regime. Specifically speaking, they respect the logic of contractual accountability while creating space for professional judgment, thereby improving the conditions under which attitudinal professionalism translates into high-quality service.

## Limitations and future research

Several limitations bound inference and set priorities for future research. First, the study is cross-sectional, and thus temporal ordering cannot be established and all estimates are interpreted as associations rather than causal effects. Stronger designs, such as panel data tracking cohorts from pre-service education through early employment, or quasi-experimental evaluations of contract-regime changes and supervision model pilots, are needed to test sequencing and identify mechanisms by which organizational embedding shapes attitudinal facets. Second, the sampling frame centers on developed metropolitan systems—Guangzhou, Shenzhen, and Shanghai—where purchase-of-service regimes are mature and administrative embedding is pronounced. External validity is therefore bounded to similar urban contexts. Site-specific non-response in organizational participation, particular in one city, may also have introduced unobserved differences. Replication in inland and county-level systems, as well as in rural settings with alternative governance mixes, would clarify portability across institutional environments and help detect whether the value-discretion profile is contingent on specific contracting logics. Third, organizational embedding is operationalized with a single indicator, that of organizational dependence on government purchase of services. We did not directly measure managerial exposure (e.g., KPI intensity, degree of procedural standardization, audit frequency, supervision style, caseload pressure). This constraints adjudication among competing mechanisms. Future studies should incorporate direct organizational measures and adopt multilevel models that link individual attitudes to agency policies and city-level contracting regimes, allowing tests of cross-level conditioning that mirror the layered framework motivating this study. Fourth, model explanatory power is modest, which is typical for multi-determinant attitudinal constructs influenced by heterogeneous individual, organizational, and contextual factors (Aiken and West, 1991; Cohen et al., 2013; Falk and Heckman, 2009). Explanatory leverage could be improved by representing latent facets and their interrelations with structural equation modeling. Specifically, using profile-based approaches (e.g., latent profile analysis) to examine how the five dimensions co-occur and whether value-discretion divergence clusters within particular organizational niches, and integrating behavioral indicators or supervisor rating to triangulate attitudinal scores. Mixed-methods designs that pair surveys with qualitative observation or interview-based process tracing would also illuminate how supervision, peer cultures, and contracting practices translate macro policy expansion and meso-level managerial governance into micro-level orientations.

Taken together, these limitations do not undermine the central contribution that clarifies and operationalizes the attitudinal facet of professionalism within an administratively embedded regime, but they do underline the need for longitudinal, multilevel, and mechanism-sensitive research capable of linking micro orientation to the organizational and policy levers identified in this study.

## Data Availability

The raw data supporting the conclusions of this article will be made available by the authors, without undue reservation.

## Appendix

The following data cleaning procedures were employed to ensure the quality and validity of the analysis. Firstly, questionnaire with over 10% of data missing were excluded from the final dataset. Secondly, for the remaining cases, missing values on scale items were handled using mean imputation for each sub-scale. For categorical variables, missing data were handled using mode imputation. Thirdly, we screened for univariate and multivariate outliers. Specifically, responses with straight-lining or abnormally short completion times were flagged and removed. Lastly, scale scores for the five dimensions of attitudinal professionalism were calculated by averaging their respective items after confirming adequate internal consistency (Cronbach's α > 0.70). After these procedures, the final analytic sample consisted of 667 respondents.
